# Reliability and Validity of the Korean Version of the Social Justice Scale in Nursing Students

**DOI:** 10.3390/ijerph192114443

**Published:** 2022-11-04

**Authors:** Hogi Jung, Yaki Yang

**Affiliations:** 1 College of General Education, Woosuk University, Wanju-gun 27841, Korea; 2 Department of Nursing, Wonkwang University, Iksan 54538, Korea

**Keywords:** justice, reliability and validity, nursing students

## Abstract

The purpose of this study was to translate and adapt a scale to evaluate social justice in nursing students and to examine the validity and reliability within a Korean context. With a survey design, a total of 267 nursing students were recruited, and data were collected using a self-administered study questionnaire to measure their levels of SJS. Using SPSS/WIN 28.0, construct validity, item convergent and discriminant validity, concurrent validity, and internal consistency reliability of the scale was evaluated. Exploratory factor analysis supported the construct validity with a four-factor solution; that explained 55.52% of the total variance. Criterion validity was demonstrated with the Social Issues Advocacy Scale (r = 0.78, *p* < 0.001). Cronbach’s α coefficient for the scale was 0.96. The findings show satisfactory construct and criterion validity and reliability of the Korean version of the SJS for measuring social justice in nursing students.

## 1. Introduction

Social justice is the foundation of public health and refers to academic and professional behaviors that can change the social values, structures, policies, and practices to give socially marginalized groups easier access to resources that can help increase their level of self-determination [1,2]. While it can be viewed as altruistic helping behavior in a broader sense, it can be differentiated from simple helping behavior based on the fact that it includes the concept of social inequality and discrimination due to power relations. Despite increased life expectancy and better health status throughout the world, there is still no improvement in health disparity according to the level of social vulnerability [3]. Health inequity may be determined by various environmental, political, economic, and social conditions that people face. Therefore, helping to give fair and equal access to medical resources, rights, and treatments to groups marginalized within society due to race, age, socioeconomic status, physical ability, and sexual orientation could also be viewed as a form of social justice [4]. Definitions of social justice include the belief that structural or social inequality must be minimized and efforts must be made to strengthen the competencies of the disadvantaged and underprivileged, and thus, practical actions of each individual present important implications for the realization of social justice [5,6].

Social justice has been an issue ever since mankind began to take part in community living. Exploitation and distribution problems emerged as humans formed communities with the division of labor and the creation of classes. Consequently, social justice began to be mentioned in religious scriptures, and after the Roman Catholic Church used the concept of social justice as a core doctrine regarding political, economic, and social poverty or inequalities in the social system, various fields beyond religion began to take an interest in social justice. In nursing, social justice includes the fair distribution of health-related resources, such as clean air, prenatal care, and competent healthcare personnel [7]. Moreover, as an issue and a right in the lives of all members of society and a value ideology pursued by nurses [7,8], it represents a fair social structure and distribution for laying the foundation for human rights and health rights [9]. The Korean Code of Ethics for Nurses clearly states the responsibilities of nursing professionals who embody justice and trust as providing equitable care while advocating human dignity and fundamental rights [10]. Accordingly, nurses have the responsibility to serve to enhance social justice through dynamic and sustained leadership in public and political decision-making for healthcare-related issues [11], while they must also promote social justice through social support for dignified life and death [12] and provide intervention plans for resolving factors that have negative health impact [13].

Social justice is presented not only as a responsibility and requirement for nursing professionals, but it is also depicted as various concepts regarding health equity, fair distribution of medical resources, human rights and health rights, professional competency, nursing practice, and social participation behavior [9,14,15,16]. For nurses, social justice could also include responsibilities and roles as a democratic citizens for resolving social determinants of health; fairly establishing human rights, freedom, and health rights; professional commitment; social participation behavior; and health equity.

Recently, educational institutions for medical and nursing professionals have presented social justice as a core value of healthcare professionals while emphasizing the ethical, moral, and educational obligations to promote social justice expected from professional nursing at national and global levels. The Institute of Medicine proposed values for promoting social justice that nursing organizations, which are at the center of healthcare, should teach to future nurses to enable them to demonstrate leadership when providing high-quality nursing accessible to everyone through the improvement of the healthcare system [17]. The American Association of Colleges of Nursing suggested social justice as a core value in undergraduate nursing education while emphasizing the need for education based on social justice that adheres to moral, legal, and humanistic principles, which must be reflected in professional nursing practice to give equal access to high-quality healthcare services as mandated by law [18,19]. Moreover, the need for a change from the existing nursing curriculum to higher education with the incorporation of social justice was proposed for responding to new demands in nursing education, along with changes in the social environment [20].

Social justice nursing education can prepare nurses to become civic nurses by strengthening the leadership and civic competencies that nursing students need in a healthcare environment so that they may practice social justice to create actual changes in communities and clinical settings [21,22]. For this, measures for integrating core social justice competencies into existing nursing curricula must be explored, while there is the need for a teaching-learning paradigm that can enhance the social justice competencies of nursing students, who are learners, by developing and applying for various social justice nursing education programs. However, despite the importance of education for strengthening social justice competencies in the field of nursing education, there is very little effort to develop social justice as a core competency in the undergraduate curriculum in Korea. There is a need to teach social justice starting from undergraduate courses so that nursing students, who are preparing to become future professional nurses, can use social justice competencies when they practice nursing as professionals in clinical settings [23].

First, a theoretical, conceptual framework for social justice must be established [24,25], and various studies related to social justice in theory and practice must be conducted to enable social justice to develop into a core nursing competency in clinical practice.

To systematically understand and study social justice competencies, a valid and reliable scale that can hold the meaning of competency is needed. This is because having a scale for a certain concept is a fundamental factor that enables quantitative understanding and systematic discussion of that concept. However, an appropriate tool for measuring social justice in nursing students has not been developed in Korea. Accordingly, the present study aimed to accurately measure the level of social justice among nursing students by revising the scale developed by Torres-Harding et al. [6] to reflect the nursing education and clinical setting in Korea. Moreover, the reliability and validity of the scale were tested to further validate the application of this social justice scale.

## 2. Methods

### 2.1. Research Design

This methodological study was designed to test the reliability and validity of the Social Justice Scale (SJS) originally developed by Torres-Harding et al. and subsequently revised and updated for Korean nursing students.

### 2.2. Participants

Participants in the study were students enrolled in the nursing departments of three universities located in G-city and J-Province, and students without clinical practice experience were excluded. In factor analysis, the sample size was calculated as 250 according to the criteria [26] that four times the minimum number of items or a sample number of 200 or more is desirable in consideration of the number of measurement variables, the number of factors, commonality, and factor loading. When considering a 10% withdrawal rate, a total of 280 surveys were distributed, and 270 surveys were collected. A total of 267 surveys were analyzed, excluding three surveys with incomplete responses.

### 2.3. Ethical Considerations

The author informed the participants of the purpose, procedure, and confidentiality of the study to the participants prior to data collection, and informed consent was obtained from the volunteers. The consent form specified that all personal information obtained will only be used for research purposes and that participants have the freedom to withdraw from the study at any time. This study was approved by the institutional review board (IRB) at W University (IRB No: WKIRB-202105- SB-031).

### 2.4. Research Instruments

#### 2.4.1. Social Justice

SJS is a tool with proven reliability and validity originally developed by Torres-Harding et al. [6]. This self-reporting scale consists of a total of 24 items in four subscales: social justice attitudes, perceived behavioral control, subjective norms, and behavioral intention. Each item is rated on a 7-point Likert scale (1: strongly disagree, 4: average, and 7: strongly agree), with higher total scores indicating a higher level of social justice. At the time of development, the reliability (Cronbach’s α) of the tool was 0.094, while the reliability of subscales social justice attitudes, perceived behavioral control, subjective norms, and behavioral intention was 0.95, 0.84, 0.82, and 0.88, respectively.

#### 2.4.2. Social Justice Advocacy Scale

The Social Justice Advocacy Scale (SJAS) was used to assess the criterion validity through the correlation with SJS [27]. This scale is suitable for assessing criterion validity because it has predictability for social justice and relatively high reliability and validity, while the content measured by each scale is relatively independent and unique. SJAS defines social justice advocacy as divided into four subscales: political and social advocacy, political awareness, social issue awareness, and confronting discrimination. This scale was developed as a Korean version, and its reliability and validity have been tested [28]. Accordingly, the present study used the Korean version of SJS (K-SJS) to test the criterion validity. At the time of development, the reliability (Cronbach’s α) of the tool was 0.94, while the reliability of subscales political and social advocacy, political awareness, social issue awareness, and confronting discrimination was 0.96, 0.88, 0.97, and 0.91, respectively.

### 2.5. Scale Development Process

#### 2.5.1. Translation of SJS

In the present study, the validity of the scale translation process was assured by following the recommendations for the translation of scales used when a scale developed in English is applied to a different language and culture [29]. In other words, the items in the final scale were completed by going through the processes of initial translation, expert panel review, back translation, preliminary survey, and cognitive interview.

In the first step, the authors translated the original tool from English to Korean and, interpreted by nursing faculty with expertise in English-to-Korean translation, confirmed that each item was translated faithfully according to the content of the original tool. The translated K-SJS was back-translated to English, without referring to the original English version, by a professor working in nursing who has a Ph.D. in English and is fluent in English and Korean to maintain independence between the original translator and back translator. Subsequently, differences in the choice of words and expressions were reviewed and revised through discussions among the researchers, original translator, and back translator. Only item #13 was revised, and all other items were used without any change to the meaning.

#### 2.5.2. Content Validity Testing

The expert group for verification of content validity consisted of a total of 10 people with more than 5 years of educational experience, 2 professors of nursing management, 2 professors of regional nursing, 2 professors of nursing policy, and 4 professors of the nursing department majoring in tool development. Content relevance was rated on a 4-point Likert scale (1: Not relevant at all, 2: Not relevant; 3: Relevant, and 4: Very relevant). Among scale-level contents validity index (S-CVI), S-CVI/Average value of 0.93–0.97 and cut-off of ≥0.90 [30] were satisfied, and as a result, 24 items comprised the final version of SJS.

#### 2.5.3. Preliminary Survey

In order to increase the content understanding and clarity of the items, a self-reporting questionnaire survey was conducted on 10 nursing students who had not seen the scale before. This preliminary survey was used to check for items that are difficult to understand and to measure the time required to complete the survey using this scale. The preliminary survey results showed that the time required to complete the survey was approximately 10–15 min. Subsequently, the main survey was conducted by completing the final version of the scale by revising words and sentences that were unclear or difficult to understand.

#### 2.5.4. Data Collection

The data used for the analysis conducted in the present study were collected during the period from 18 May 2021 to 13 July 2021. The self-administered questionnaire was used to collect the data. Before the collection of the data, the objective and procedure of the study were explained to the subjects, and the questionnaire was distributed, along with a written consent form and envelope. The written consent forms were sealed in envelopes by the subjects and submitted separately. All the data from the questionnaire were processed anonymously.

#### 2.5.5. Statistical Analysis

The collected data were analyzed using the SPSS Statistics 28.0 program. The participants’ general characteristics were examined using descriptive statistics. For item analysis of the tool, correlation coefficients between individual item scores and total scores were calculated. The verification of the construct validity of the preliminary scale was calculated by exploratory factor analysis. Kaiser-Meyer-Olkin (KMO) values were calculated, and Bartlett’s sphericity was verified to determine the possibility of exploratory factor analysis for verification of construct validity. As an exploratory factor analysis method, the main axis factor extraction method and the Verimax rotation method were used, and the factors were extracted based on the eigen value of 1.00 or higher, and the factor load was applied to the standard >0.40

In order to test the convergent validity and discriminant validity of each item, a multi-traitmulti-item matrix analysis was performed. The criterion validity was analyzed using Pearson’s correlation coefficients. The reliability of the scale was tested based on Cronbach’s α for internal consistency.

## 3. Results

### 3.1. General Characteristics of the Study Population

The characteristics of the participants are listed in Table 1. The study population consisted of 258 females (96.6%), including 154 juniors (57.7%). With respect to having a religion, 172 participants (64.4%) responded “No.” With respect to the reason for choosing the major, 135 (50.6%) participants indicated “ease of employment.” With respect to grade point average (GPA) in the previous semester, 78 (29.2%), 119 (44.2%), and 71 (26.6%) participants reported >4.0, 3.5–4.0, and <3.5, respectively. With respect to satisfaction with the major, 171 (64%) participants indicated “satisfied.” With respect to how long they wished to stay in nursing, 123 (46.1%) participants reported “as long as possible,” With respect to experience in social justice-related education, 202 participants (75.7%) indicated “No” (Table 1).

### 3.2. Item Analysis

The results of item analysis for assessment of items in the scale showed a mean score of 3.85 ± 0.45 points (5-point scale). For the correlation coefficient between individual items and all items, deletion is recommended for those with <0.30 due to low contribution and ≥0.80 due to duplicates [31]. In the present study, the mean correlation coefficient between the initial 24 items and all items was 0.65, with a range of 0.51 to 0.78. Therefore, specific items were not excluded since the items were not <0.30 or ≥0.80.

### 3.3. Validity Analysis

#### 3.3.1. Construct Validity

KMO and Bartlett’s test of sphericity was performed to determine the sampling adequacy in the factor analysis for testing the construct validity of the scale. The results showed KMO = 0.88 and ×2 = 2885.40 (*p* < 0.001), and thus, the factor analysis model was determined to be adequate. Principal factor analysis was used for factor extraction, while varimax rotation was used for factor rotation. In the first factor analysis, a total of four factors with Eigen value ≥ 1 was extracted. The final number of items for the scale was determined to be 24. Based on the factor analysis results, the first, second, third, and fourth factors were identified to be social justice attitudes, perceived behavioral control, subjective norms, and behavioral intention, respectively, and each other test factor had an explanatory power of 16.89%, 16.31%, 13.04%, and 9.28%, respectively, with a total cumulative explanatory power of 55.52% (Table 2). When considering the conceptual meaning of the scale, the first, second, third, and fourth factors were named social justice attitudes, perceived behavioral control, subjective norms, and behavioral intention, respectively.

#### 3.3.2. Convergent Validity and Discriminant Validity

The convergent validity of an item is established when the correlation coefficient calculated after controlling the items overlapped with the subscale of each item is ≥0.40. The discriminant validity of an item is established when the correlation coefficient of the subscale that the item belongs to is larger than that of other subscales [32]. In the present study, multi-trait/multi-item matrix analysis was performed to test the convergent and discriminant validity of items (Table 3). The correlation coefficients for items and the subscales to which they belong were all ≥0.40, indicating a scaling success rate of 100% for convergent validity. Moreover, the correlation coefficients of all items for the subscale to which they belong were larger than that of other subscales, indicating a scaling success rate of 100% for discriminant validity.

#### 3.3.3. Criterion Validity

In order to test the criterion validity of the scale, the correlations among the four subscales of SJAS by Lim (28) were investigated (Table 4). The results showed a positive correlation between social justice and social justice advocacy (r = 0.78, *p* < 0.001). The entire scale for social justice also showed positive correlations with subscales of political and social advocacy (r = 0.68, *p* < 0.001), political awareness (r = 0.65, *p* < 0.001), social issue awareness (r = 0.66, *p* < 0.001), and confronting discrimination (r = 0.50, *p* < 0.001). Moreover, the subscales of SJS showed positive correlations with the subscales of SJAS.

### 3.4. Reliability

Internal consistency, as measured with Cronbach’s α, was 0.91, and Cronbach’s α for each subcategory was as follows: social justice attitudes 0.838, perceived behavioral control 0.803, subjective norms 0.808, and behavioral intention 0.835 (Table 2).

## 4. Discussion

The present study aimed to test the validity and reliability of SJS by Torres-Harding et al. for nursing students in Korea. The study attempted to increase the external validity by recruiting juniors and seniors enrolled in nursing programs at three colleges in J city. As future clinical nurses, nursing students must practice nursing as professionals through social justice competencies in clinical practice [23]. The present study was significant in that it was attempted under the current situation in Korea, where discussions on the moral values and roles of social justice in the field of nursing are lacking.

SJS by Torres-Harding et al. was developed based on the concepts of four factors (social justice attitudes, perceived behavioral control, subjective norms, and behavioral intention) related to social justice with the conceptual framework of the theory of planned behavior by Ajzen. The theory of planned behavior by Ajzen is useful for explaining various human behaviors, and just as the theory of planned behavior assumes, many studies have demonstrated that attitudes, subjective norms, and perceived behavioral control can very accurately predict intention and that intention and perceived behavioral control can explain a significant portion of behavioral changes [33].

In the study, K-SJS for nursing students was developed with 24 items in four subscales of social justice attitudes, perceived behavioral control, subjective norms, and behavioral intention, with each item rated on a 5-point Likert scale.

The first factor, social justice attitudes, consisted of 11 items, and this factor had the highest explanatory power of 16.89% for the social justice of nursing students. Social justice attitudes represent specific beliefs that individuals perceive in relation to the performance of social justice activities. This can be divided into personal (e.g., self-perception and monitoring), interpersonal (e.g., educating other people on social inequality or encouraging people to participate in activities), community (e.g., assessing the special needs of the community or developing community volunteer programs), and institutional/political (e.g., confronting discriminatory policies or practices) dimensions. To practice social justice as nurses, they must be not only aware of the existence of inequality but also be able to recognize that unfair conditions are due to the institutional oppression of certain groups in our society. Moreover, they must also recognize that institutional barriers, policies, and laws influence such injustice and harm. However, they must not focus solely on the disproportionate characteristics of a specific group or a specific individual within a group. In a study by Torres-Harding et al., all four subscales showed high correlations with the public service motivation scale, which suggested that individuals who give positive responses to the items in SJS are likely to show more interest in public service jobs. This is also consistent with the ideals of social justice, reflecting the fact that people who wish to practice social justice may prioritize jobs that serve others [34].

In a study by Arthur et al. [35] on social justice competency and career development, supportive attitudes, respect, empathy, and passionate attitudes for promoting social justice were emphasized as specific attributes needed for career development experts to practice social justice. In other words, actively taking part in the pain of others who emotionally face discrimination and inequality can further strengthen the practice of social justice than cognitively recognizing inequitable situations and understanding the positions of others. Therefore, attitude towards social justice could be an important variable for predicting behavioral intention for social justice. Based on the study that examined changes in social justice attitudes of nursing students after simulation learning on the experience of difficult financial, employment, and parenting situations faced by poor families [36], it is necessary to set the social justice-related program learning outcomes and operate the curriculum for positive cultivation of social justice attitudes among nursing students.

The second factor, perceived behavioral control, had an explanatory power of 16.31%, and it consisted of five items, including items related to self-assessment of how much change is possible or whether the existing situation can be changed. Perceived behavioral control is a major predictor of behavioral intention, and when the behavior itself is especially difficult or challenging, it can directly predict the behavior [33]. Recently, in Korea, the enactment of the Nursing Act has been promoted to ensure patient safety, as well as to secure the work stability and professionalism of nurses, and provide better health and medical services based on collaboration among medical workers. However, due to conflicts of interest among medical groups, it has been in conflict for years and has not been enacted. Heightened perceived behavioral control can directly influence social justice practice. It can also create the expectation that the outcomes of practicing social justice would be positive and promote interest in social justice activities, which can lead to social justice practice.

The third factor, subjective norms, had an explanatory power of 13.04%, and it consisted of five items related to how an individual perceives the patterns of likes, dislikes, and indifferences related to social justice activities of those around him or her. Subjective norms refer to personal perception of what opinion an individual has about specific behaviors of those close to him or her (e.g., friends, family, peers, etc.) [33]. In other words, the thoughts and opinions people close to them, who mutually influence each other, have about specific behavior are a very important and stronger motivation to respond to such opinions, increasing the likelihood of performing such behavior [37]. Miller et al. [38] analyzed the social justice behavior prediction pathway model to confirm that interest in social justice and self-efficacy are direct predictors of social justice practice. Interest and self-efficacy in the social justice behavior prediction pathway model are conceptually similar to subjective norms among the subscales of SJS.

The type of relationship a person forms with another person could influence the way the person perceives a message [39], but the present study did not include other normative factors, such as perception, relationships, broader social context, and media. Therefore, additional studies with the inclusion of other items, such as laws, policies, other social norms, and messages within the surrounding environment that can influence individuals, are needed.

The fourth factor, behavioral intention, had an explanatory power of 9.28%, and it consisted of five items on choices related to social justice activities that the individual planned, agreed on goals, and specific performance. Goals help organize and maintain the long-term behaviors of individuals, even if they are not readily visible, while they also motivate other goals and actions [40]. The model by Ajzen considers intention as a major predictor of behavior, more so than attitudes or subjective norms, which is similar to another study reporting that a positive social justice attitude is a major predictor of behavioral change [41].

The identity of nursing professionals is an important influencing factor in building social justice competency, which is one of the core values of professionals [42], and thus, it is necessary to provide education to improve the identity of professionals starting from the undergraduate curriculum to strengthen social justice competency. In a study on 1610 women, identity as a woman activist was identified as the most powerful predictor of being a feminist or being actually active in gender-related problems [43]. In future studies on nursing students, the correlation between intention to practice social justice and identity as a nursing professional should be investigated, and other variables, such as professional identity, should also be considered for predicting social justice-related behaviors.

The criterion validity of the final scale revised and developed through the present study was tested against SJAS [28]. Criterion validity testing against SJAS showed a highly positive correlation (r = 0.78, *p* < 0.001). Based on the result, it was determined that the scale developed in the present study is a valid scale for measuring the social justice competencies of nursing students. With respect to correlations by each factor, political and social advocacy and political awareness showed higher correlations with behavioral intention in SJS (0.72 and 0.65, respectively). A high correlation between political and social advocacy and behavioral intention supported the model by Ajzen, which showed that intention was a major predictor of action, more so than attitudes or subjective norms. A high correlation between political awareness and behavioral intention supported the analysis of the social justice behavior prediction pathway model by Miller et al. [38], which confirmed that interest in social justice is a direct predictor of social justice practice.

Reliability test results showed high reliability with Cronbach’s α of 0.91. The reliability of subscales was also high, ranging between 0.80 and 0.84. Therefore, the scale could be valuable when used in actual nursing education.

In a previous study, attitudes, beliefs, knowledge, skills, and behaviors of college students were identified as key concepts that influence social justice-related core competencies [44], and concepts such as perception and advocacy skills were reported to be closely correlated [45]. Moreover, a study on multicultural education for social justice [46] identified behavioral intention as an influencing factor of social justice competency, while a study on social justice nursing education strategy [36] identified attitudes, behaviors, social norms, and critical thinking as very important competencies for social justice practice. The behavioral intention could be viewed as a natural outcome that can be induced by enhancing attitudes, subjective norms, and perceived behavioral control, and thus, educational programs for social justice attitudes, subjective norms, and perceived behavioral control must be specifically designed and accompanying teaching-learning methods and strategies must be prepared to enhance social justice among nursing students.

K-SJS, which was tested for reliability and validity in the present study, was revised by checking the cultural validity of Korea, and it consisted of four subscales for social justice among nursing students. Understanding the cognitive process that leads to each individual participating in social justice-related activities could be helpful in developing educational programs for enhancing social justice competencies and testing the effects of such programs. Moreover, a more accurate assessment and scientific prediction of factors that contribute to social justice could be helpful for establishing measures to promote social justice and develop follow-up action plans.

## 5. Conclusions

Social justice is an essential practical competency that clinical nurses must have to provide the best possible care to patients. In the present study, the validity and reliability of a scale can measure the social justice competencies of nursing students with consideration of the clinical setting and nursing education situation in Korea. The significance of the present study is that it provides the theoretical evidence for the development of educational and training programs for enhancing social justice by developing a scale that reflects the characteristics of Korea and can identify the attributes of social justice among nursing students when there is a lack of systematic education and measurement tools for social justice of nurses. The scale developed by the present study is expected to be used for education, training, and testing of the effects of relevant interventions for social justice among nursing students and clinical nurses.

## 6. Limitations

In the preliminary survey, the number of students was 10, and students from other courses were not included.

## Figures and Tables

**Table 1:** Demographic Characteristics of Participants (*N* = 267).

Characteristics	Categories	N(%)
Gender	Male	9 (3.4)
	Female	258 (96.6)
School year	Junior	154 (57.7)
	Senior	113 (42.3)
Religion	Yes	95 (35.6)
	No	172 (64.4)
Motivation for major selection	Aptitude	86 (32.2)
	Ease of employment	135 (50.6)
	Suggestion around	30 (11.2)
	Highschool grade	16 (6.0)
Academic record	>4.0	78 (29.2)
	3.5–4.0	119 (44.2)
	<3.5	71 (26.6)
Major Satisfaction	Satisfied	171 (64.0)
	Usually	88 (33.0)
	Unsatisfied	8 ( 3.0)
Duration of Work	As long as possible	123 (46.1)
	Required time only	98 (36.7)
	Resign quickly	10 (3.7)
	Not sure	36 (13.5)
Experience of class regarding Social Justice	Yes	65 (24.3)
	No	202 (75.7)

**Table 2:** Factor Analysis of Social Justice Scale (*N* = 267).

Factors	Items		M ± SD	Factor Loading			
				1	2	3	4
Social justice: attitudes	item 5	I believe that it is important to help individuals and groups to pursue their chosen goals in life.	4.34 ± 0.65	0.799	0.073	0.111	0.266
	item 9	I believe that it is important to support community organizations and institutions that help individuals and groups achieve their aims.	4.12 ± 0.70	0.780	0.010	0.100	0.408
	item 10	I believe that it is important to promote fair and equitable allocation of bargaining powers, obligations, and resources in our society.	4.05 ± 0.79	0.754	0.055	0.247	0.080
	item 6	I believe that it is important to promote the physical and emotional well-being of individuals and groups.	4.45 ± 0.63	0.721	−0.134	0.174	0.281
	item 4	I believe that it is important to try to change larger social conditions that cause individual suffering and impede well-being.	4.39 ± 0.66	0.703	0.040	0.109	0.049
	item 7	I believe that it is important to respect and appreciate people’s diverse social identities.	4.42 ± 0.64	0.697	0.012	0.095	0.194
	item 1	I believe that it is important to make sure that al individuals and groups have a chance to speak and be heard, especially those from traditionally ignored or marginalized groups.	4.30 ± 0.59	0.617	0.182	−0.099	−0.007
	item 11	I believe that it is important to act for social justice.	4.08 ± 0.69	0.591	0.235	0.004	0.357
	item 8	I believe that it is important to allow others to have meaningful input into decisions affecting their lives.	3.73 ± 0.85	0.549	0.085	0.251	0.070
	item 2	I believe that it is important to allow individuals and groups to define and describe their problems, experiences, and goals in their own terms.	4.12 ± 0.61	0.541	0.129	0.081	0.172
	item 3	I believe that it is important to talk to others about societal systems of power, privilege, and oppression.	4.04 ± 0.83	0.447	0.394	0.007	−0.118
Social justice: perceived behavioral control	item 18	Other people around me felt that it is important to engage in dialogue around societal injustices.	3.38 ± 0.96	0.029	0.771	0.258	0.190
	item 19	Other people around me are supportive of efforts that promote social justice.	3.47 ± 0.87	0.224	0.727	0.163	0.327
	item 17	Other people around me are engaged in activities that address social justice issues.	2.93 ± 1.02	−0.096	0.696	0.268	0.190
	item 20	Other people around me are aware of issues of social injustices and power inequalities in our society.	3.68 ± 0.86	0.000	0.693	0.078	0.076
	item 14	If I chose to do so, I am capable of influencing others to promote fairness and equality.	3.57 ± 0.82	0.158	0.736	0.188	0.150
Social justice: subjective norms	item 16	I am certain that if I try, I can have a positive impact on my community	3.44 ± 0.84	0.065	0.198	0.713	0.267
	item 13	I am certain that I poses an ability to work with individuals and groups in ways that are empowering.	3.70 ± 0.82	0.188	0.081	0.701	−0.005
	item 12	I am confident that I can have a positive impact on others’ lives.	3.65 ± 0.83	−0.003	0.156	0.678	0.099
	item 15	I feel confident in my ability to talk to others about social injustices and the impact of social conditions on health and well-being.	3.38 ± 0.89	0.020	0.388	0.554	0.210
Social justice: behavioral intentions	item 23	In the future, I intend to engage in activities that will promote social justice.	3.74 ± 0.85	0.207	0.232	0.077	0.746
	item 2	In the future, I will do my best to ensure that all individuals and groups in my community have a chance to speak and be heard.	3.76 ± 0.85	0.283	0.274	0.277	0.665
	item 22	In the future, I intend to talk with others about social power inequalities, social injustices, and the impact of social forces on health and well-being.	3.73 ± 0.88	0.133	0.464	0.204	0.644
	item 2	In the future, I intend to work collaboratively with others so that they can define their own problems and build their own capacity to solve problems.	3.81 ± 0.77	0.264	0.126	0.245	0.625
Eigen value				7.28	2.68	1.58	1.28
Explained variance (%)				16.89	16.31	13.04	9.28
Accumulative variance (%)				16.89	33.20	46.24	55.52
KMO = 0.88, Bartlett’s test: χ^2^ = 2885.40 (*p* < 0.001)							
Cronbach’s α				0.838	0.803	0.808	0.835
Total Cronbach’s α = 906							

**Table 3:** Multi-trait/Multi-item Matrix Analysis (*N* = 267).

Factors	Items	F1	F2	F3	F4	2 Standard Error
		r	r	r	r	
Social justice: attitudes	1	0.688	0.162	0.196	0.294	0.073
	2	0.644	0.305	0.214	0.418	0.075
	3	0.598	0.218	0.305	0.306	0.101
	4	0.623	0.194	0.130	0.297	0.081
	5	0.691	0.268	0.211	0.446	0.079
	6	0.654	0.255	0.081	0.376	0.077
	7	0.690	0.237	0.163	0.391	0.078
	8	0.590	0.379	0.231	0.269	0.104
	9	0.595	0.324	0.256	0.449	0.085
	10	0.620	0.349	0.236	0.309	0.097
	11	0.591	0.260	0.344	0.446	0.085
Social justice: perceived behavioral control	12	0.231	0.725	0.301	0.349	0.102
	13	0.378	0.715	0.290	0.332	0.100
	14	0.306	0.764	0.384	0.413	0.100
	15	0.364	0.737	0.524	0.442	0.109
	16	0.353	0.799	0.450	0.461	0.103
Social justice: subjective norms	17	0.249	0.464	0.821	0.427	0.125
	18	0.268	0.462	0.854	0.468	0.117
	19	0.400	0.425	0.806	0.584	0.106
	20	0.217	0.314	0.705	0.347	0.106
Social justice: behavioral intentions	21	0.501	0.496	0.472	0.832	0.103
	22	0.390	0.471	0.575	0.833	0.108
	23	0.476	0.351	0.426	0.820	0.104
	24	0.437	0.435	0.384	0.788	0.095

**Table 4:** Correlation between K-SJS and Social Justice Advocacy Scale (*N* = 267).

		Social Justice Advocacy Scale				
		Total	①	②	③	④
		r (p)				
K-SJS	Total	0.783 **	0.681 **	0.651 **	0.660 **	0.503 **
	⑤	0.603 **	0.456 **	0.542 **	0.620 **	0.413 **
	⑥	0.561 **	0.508 **	0.445 **	0.448 **	0.370 **
	⑦	0.537 **	0.527 **	0.411 **	0.386 **	0.282 **
	⑧	0.793 **	0.721 **	0.653 **	0.577 **	0.525 **

** *p* < 0.001 ① Political and Social Advocacy ② Political Awareness ③ Social Issue Awareness ④ Confronting Discrimination ⑤ Social justice: attitudes ⑥ Social justice: perceived behavioral control ⑦ Social justice: subjective norms ⑧ Social justice: behavioral intentions.

## Data Availability

After acceptance of this article, data used for data analysis will be made publicly available.
